# The Multiple Faces of the GH/IGF Axis

**DOI:** 10.3390/cells11020217

**Published:** 2022-01-10

**Authors:** Vera Chesnokova

**Affiliations:** Pituitary Center, Department of Medicine, Cedars-Sinai Medical Center, Los Angeles, CA 90048, USA; Vera.Chesnokova@cshs.org

Over the past two decades, interest in the role of the somatotroph growth hormone/insulin-like growth factor (GH/IGF1) axis in multiple aspects of physiology and pathology has grown exponentially. In addition to its well-described role in somatic growth and development, a role for the GH/IGF1 axis has been described in metabolism, bone, muscle, and lung homeostasis, inflammatory response, age-associated physiological and pathological changes, neoplastic development and chemotherapy resistance. In this Special Issue of *Cells*, six reviews and four experimental papers address emerging areas of GH/IGF1 axis action across a variety of biological processes, including central and peripheral metabolic pathways, bone health and development, muscle endurance, hyperoxia-induced injury, genomic changes, and aging.

GH plays an important role in regulating metabolic function. Three reviews consider the central and peripheral effects of GH on metabolism, as well as the mechanisms underlying the feedback effects of nutrients on the somatotroph axis.

It has long been assumed that the metabolic effects of GH are mediated by its classical target tissues of liver, adipose, and muscle. However, as discussed in the review by Donato et al. [1], a growing body of evidence now indicates GH’s role of central regulation of different aspects of metabolism. A wide array of GH-responsive neurons was discovered in different areas of the brain, and are particularly abundant in hypothalamic nuclei controlling metabolism. The experimental results described in this review showing that central GH action modulates insulin sensitivity, glucose homeostasis, and metabolic response to calorie restriction strongly support the involvement of GH in central regulation of energy and glucose homeostasis.

A very exhaustive review on the regulation of metabolism by GH/growth hormone receptor (GHR) in the liver is presented by Vazques-Borrego et al. [2]. While evidence indicates that GH controls hepatocyte metabolism indirectly through lipid mobilization from white adipose tissue to the liver as well as through modulation of IGF1 production and function, this review also describes the direct effects of GH/GHR on substrate metabolism. The authors present data on molecular mechanisms of GH action on hepatocyte carbohydrate and lipid metabolism, and discuss the effects of GH/IGF1 axis within the context of fasting or food excess, duration and severity of food deprivation, time of day, and age. Important questions about the limitations of in vitro experimental models are addressed, and the authors critically discuss both the power and the limitations of genetically modified experimental mouse models in studying the role of GH in metabolic health and disease.

In line with this body of work, the comprehensive review by Caputo et al. [3] is focused on the less-recognized role of nutrients in the regulation of GH and GH signaling, summarizing the mechanisms of somatotroph axis activation by macro- and micro-nutrients, as well as GH action on nutrient utilization. Specific roles of macronutrients (carbohydrates, fiber, amino acids and proteins, lipids and free fatty acids) and micronutrients (vitamins and minerals) are discussed, as are the specific effects of different types of diets, including calorie restriction, suggesting that balanced nutrition is essential for GH/IGF1 homeostasis. 

The GH/IGF1 axis plays an important role in postnatal growth and development. While GH/IGF1 endocrine and paracrine action in bone is well documented, the review by Koffi et al. [4] describes a lesser-investigated role of this axis in craniofacial bones and the dento-alveolar complex in cells, experimental animals, and human patients. After presenting in vivo and in vitro data on the mechanisms of action in normal and pathological states in various experimental models, the authors conclude that the GH/IGF1 axis is necessary for the development of the dento-alveolar complex as well as for oral bone repair and regeneration, and that the addition of growth factors such as transforming growth factor β (TGFβ) and platelet-derived growth factor (PDGF) may counteract oral bone resorption. 

GH/IGF1 is centrally regulated by hypothalamic GH releasing hormone (GHRH), while GHRH and GHRH receptors are also expressed in peripheral tissue. The specific role of GHRH in the lung is discussed in the review by Zhang et al. [5], which describes mechanisms of GHRH action on gene expression, mitochondria respiration, and intracellular signaling in lung development, growth, and repair in both cellular and animal models. The authors compare the effects of synthetic peptides mimicking GHRH action with endogenous GHRH, and analyze the effects of GHRH antagonists, which exert growth inhibitory effects in lung cancer cells in vitro and in vivo and act as anti-inflammatory agents. The authors further evaluate new findings on the role of GHRH in lung inflammation, fibrosis, and cancer and comprehensively review both current challenges and opportunities for potential clinical application of GHRH antagonists in lung disease. 

The intriguing question of why Laron syndrome (LS) patients carrying a GH receptor (GHR) mutation do not develop cancer is considered in the paper by Werner et al. [6], which reviews studies of genome-wide profiling of LS patients. These patients exhibit very high circulating levels of GH, while IGF1 is significantly suppressed. Using a computational approach, it was demonstrated that genes involved in the control of the cell cycle, motility, growth, and oncogenic transformation are downregulated in LS patient lymphoblastoid cells, while genes associated with protection from toxic xenobiotic substances and metabolites are induced. The potential roles of several differentially expressed genes in LS are discussed. Combining experimental and computational approaches, the authors reach an important conclusion that the life-long lack of exposure to IGF1 in LS patients has a protective effect by enhancing apoptosis, autophagy, and responses to oxidative stress.

Several important findings related to the role of GH/IGF1 are described in the four experimental papers included in this Special Issue. In line with the data presented in the review by Donato et al. [1], two experimental papers describe the central role of GH signaling in metabolism. In the first paper, Bezerra Medeiros de Lima et al. [7] show the role of the GHR/SIRT1 axis in fasting. The authors show that in the arcuate nucleus of the hypothalamus (ARC), a region that regulates feeding and whole-body energy homeostasis, GHR is co-expressed with SIRT1, a deacetylase that controls lifespan and senses nutrient availability. Importantly, SIRT1 is induced in GHR-expressing neurons in response to fasting, and its expression is attenuated in neurons lacking GHR. Considering the anti-aging effects of SIRT1 and increased lifespan in GHR knockout mice, these results imply a novel role for SIRT1 within the somatotroph axis in response not only to fasting but potentially also the aging process. 

The second experimental paper from the same group [8] examines the role of GHR-expressing neurons in ARC. The authors generated a novel transgenic mouse that overexpresses GHR in ARC (ARC^GHR+^) and used the DREADD (designer receptor exclusively activated by designer drug) technique to control ARC^GHR+^ neuronal activity. Activation of these neurons increased glucose turnover and whole-body glycolysis, and specifically activated glycolysis and insulin sensitivity in skeletal muscle, indicating a novel metabolic regulatory pathway through GH-responsive GHR in the ARC. 

Human GH is used by some athletes as a performance-enhancing drug, despite its illegality. Brenmoehl et al. [9] examined the physiological role of pituitary GH in skeletal muscle in DUhTP “marathon” mice selected for high running performance compared to sedentary DUhTP mice to explore GH/IGF1 axis action in skeletal muscle during exercise. After 3 weeks of endurance exercise, pituitary Pou1 (Pit1), a pituitary-specific transcription factor for GH, as well as mTORC1 and mTORC2 pathways were suppressed, with downregulation of multiple GH/IGF1 signaling compounds in muscle including Ghr, Igf1, Igf1r, Irs1 and 2, Akt3, Rictor, Rptor, and others. The results suggest that the central downregulation of GH/IGF1 and peripheral downregulation of mTORC activity represent adaptive responses for high running performance in DUhTP mice, and lead to the unexpected conclusion that suppression of the somatotroph axis, not its activation, is required for improving running endurance.

Prematurely born infants often require supplemental oxygen, which can trigger a chronic form of lung injury, and multiple lines of evidence suggest that the GH/IGF1 axis is involved in the regulation and proliferation of lung cells as well as lung tissue repair. Vohlen et al. [10] investigated the regulation of somatotroph action during lung injury in newborn mice and showed a previously unrecognized uncoupling of the GH/IGF1 signaling pathway and differentially regulated GH and IGF1 functions in neonatal lung injury. Specifically, hyperoxia reduced GHR/STAT signaling while increasing IGF1 mRNA and pAKT signaling. Moreover, while GH induced the inflammatory factor interleukin 6 (IL-6), IGF1 had the opposite effect. This study proposed a novel hypothesis involving differential action of GH and IGF1 in neonatal mice exposed to short-term and prolonged hyperoxia, and suggests a potential role for IGF1 as a therapeutic option in lung recovery.

In conclusion, this Special Issue of *Cells* highlights important new advances in our understanding of the mechanisms of GH/IGF1 regulation. Across very different research areas, these studies provide some clarity on how this axis might impact a wide array of pathological and physiological conditions. We thank all of the contributors to this Special Issue of *Cells* and thank the journal’s staff members for their editorial support.
